# Aliskiren: Just a New Drug for Few Selected Patients or an Innovative Molecule Predestinated to Replace Arbs and Ace-Inhibitors?

**DOI:** 10.3390/ph2030118

**Published:** 2009-11-27

**Authors:** Salvatore Novo, Giovanni Fazio, Elena Raccuglia

**Affiliations:** Department of Internal Medicine and Cardiovascular Diseases, University Hospital “Paolo Giaccone”, University of Palermo, Italy

**Keywords:** aliskiren, RAS system, ACE inhibitors, angiotensin receptor blockers

## Abstract

The renin-angiotensin-aldosterone system (RAAS) plays a dominant role in the pathophysiology of hypertension, diabetes mellitus, chronic kidney disease and chronic heart failure. Therefore, drugs that block key components of the RAAS such as ACE inhibitors (ACEI) and angiotensin receptor blockers (ARBs) have gained wide clinical use for these indications. Despite progress, the morbidity and mortality of patients treated with ACEI or ARBs remain high. Aliskiren (Tekturna, Rasilez) is the first orally active inhibitor of renin approved for clinical use as an antihypertensive agent. The development program has established that at the licensed doses of 150 mg and 300 mg. Aliskiren is effective either as monotherapy or in combination with drugs from the other major classes. In this review we analyze and review the information already gained with Aliskiren, raises questions regarding the advantages of DRIs as monotherapy compared to marketed ACEIs and ARBs, their potential added value in combination with other RAAS modulators and other still unproven benefits in relation to prorenin and renin receptor biology.

## Introduction

Hypertension is one of the most important risk factors for, and causes of, cardiovascular disease. The difficulty in achieving a normal blood pressure range in some patients makes the rate of cardiovascular disease high [1,2,3]. For some years renin-angiotensin system inhibitors such as angiotensin-converting enzyme inhibitors (ACEIs) and angiotensin receptor blockade (ARBs) have been objects of interest for treatment of cardiovascular disease. Aliskiren, the first approved renin inhibitor to reach the market, is a low molecular weight, orally active, hydrophilic nonpeptide molecule, which blocks angiotensin I generation. However it might also become a reasonable therapeutic choice in a broad number of clinical conditions, as stable coronary artery disease, cerebrovascular and cardiorenal disease, diabetes, and peripheral arterial disease [1,2,3,4,5].

## Clinical Pharmacokinetics and Pharmacodynamics of Aliskiren

Aliskiren is the first orally bioavailable direct renin inhibitor approved for the treatment of hypertension. It acts at the point of activation of the renin-angiotensin-aldosterone system, or renin system, inhibiting the conversion of angiotensinogen to angiotensin I by renin and thereby reducing the formation of angiotensin II by angiotensin-converting enzyme (ACE) and ACE-independent pathways. Aliskiren is a highly potent inhibitor of human renin in vitro (concentration of aliskiren that produces 50% inhibition of renin 0.6 nmol/L) [6]. Aliskiren is rapidly absorbed following oral administration, with maximum plasma concentrations reached within 1-3 hours. The absolute bioavailability of aliskiren is 2.6%. The binding of aliskiren to plasma proteins is moderate (47–51%) and is independent of the concentration. Once absorbed, aliskiren is eliminated through the hepatobiliary route as unchanged drug and, to a lesser extent, through oxidative metabolism by cytochrome P450 (CYP) 3A4. Unchanged aliskiren accounts for approximately 80% of the drug in the plasma following oral administration, indicating low exposure to metabolites. The two major oxidized metabolites of aliskiren account for less than 5% of the drug in the plasma at the time of the maximum concentration. Aliskiren excretion is almost completely via the biliary/faecal route; 0.6% of the dose is recovered in the urine. Steady-state plasma concentrations of aliskiren are reached after 7-8 days of once-daily dosing, and the blood persistence for aliskiren is approximately 2 [6]. After reaching the peak, the Aliskiren plasma concentration declines in a multiphase fashion. No clinically relevant effects of gender or race on the pharmacokinetics of aliskiren are observed, and no adjustment of the initial aliskiren dose is required for elderly patients or for patients with renal or hepatic impairment. Aliskiren showed no clinically significant increases in exposure during coadministration with a wide range of potential concomitant medications, although increases in exposure were observed with P-glycoprotein inhibitors. Aliskiren does not inhibit or induce CYP isoenzyme or P-glycoprotein activity, although aliskiren is a substrate for P-glycoprotein, which contributes to its relatively low bioavailability. Aliskiren is approved for the treatment of hypertension at once-daily doses of 150 mg and 300 mg. Phase II and III clinical studies involving over 12,000 patients with hypertension have demonstrated that aliskiren provides effective long-term blood pressure (BP) lowering with a good safety and tolerability profile at these doses [1,2,3,4,5,6,7,8]. 

### Inhibition of the Plasma renin activity

Aliskiren inhibits plasma renin activity (PRA) by up to 80% following both single and multiple oral-dose administration. Similar reductions in PRA are observed when aliskiren is administered in combination with agents that alone increase PRA, including diuretics (hydrochlorothiazide, furosemide), ACE inhibitors (ramipril) and angiotensin receptor blockers (valsartan), despite greater increases in the plasma renin concentration [9,10,11]. Moreover, PRA inhibition and BP reductions persist for 2-4 weeks after stopping treatment, which is likely to be of benefit in patients with hypertension who occasionally miss a dose of medication. Preliminary data on the antiproteinuric effects of aliskiren in type 2 diabetes mellitus suggest that renoprotective effects beyond BP lowering may be possible. Further studies to evaluate the effects of aliskiren on cardiovascular outcomes and target organ protection are ongoing and will provide important new data on the role of direct renin inhibition in the management of hypertension and other cardiovascular disease [12,13,14,15].

### Pharmacokinetic interactions of the direct renin inhibitor aliskiren with metformin, pioglitazone and fenofibrate

Hypertension and type 2 diabetes are common comorbidities, thus many patients receiving antihypertensive medication require concomitant therapy with hypoglycaemic or lipid-lowering drugs. Co-administration of aliskiren with metformin, pioglitazone or fenofibrate had no significant effect on the pharmacokinetics of these drugs in healthy volunteers. These findings indicate that aliskiren can be co-administered with metformin, pioglitazone or fenofibrate without the need for dose adjustment [2,4,13,16].

## Now That We Have a Direct Renin Inhibitor, What Should We Do With It?

Aliskiren is the first renin inhibitor to be licensed for use as an antihypertensive drug in both the United States and Europe. Opinions vary considerably concerning the future of aliskiren and renin inhibition. Some experts argue that renin inhibitors should only be prescribed when less expensive blockers of the renin-angiotensin system (RAS), with established effects on morbidity and mortality, are not tolerated or have failed to reduce blood pressure effectively. Others propose that because renin is a highly specific catalyst for the rate-limiting step of the RAS, renin inhibitors have the potential to supersede angiotensin-converting enzyme inhibitors and angiotensin receptor blockers as the preferred inhibitors of the cascade in patients with particular pathologies and/or genotypes [6]. It has also been suggested that dual blockade of the RAS might be particularly advantageous. As a first stage, aliskiren is reimbursed in Italy only for hypertensive patients not controlled with concomitant treatment with at least two antihypertensives [17]. 

## Role of Direct Renin Inhibitors in Chronic Kidney Disease

The evidence supporting the use of renin inhibitors to control hypertension, but limiting this use in patients with kidney disease progression even if ACE and ARB decrease cardiovascular risk in patients with chronic kidney disease. Renin-angiotensin-aldosterone system inhibition exerts a renoprotective effect independent of blood pressure reduction. Many studies using an end-point of proteinuria compared the effects of angiotensin-converting enzyme inhibitor or angiotensin receptor blocker monotherapy with combination ACE/ARB therapy. Despite methodological limitations, most studies suggest that combination therapy provides a greater antiproteinuric effect than monotherapy, perhaps because of more prolonged and complete RAAS inhibition. COOPERATE and ONTARGET used more robust end-points to study renoprotective effects. In COOPERATE Study; combination therapy resulted in significantly longer times to doubling serum creatinine or developing end-stage renal disease than Trandolapril or losartan monotherapy. However, a secondary ONTARGET finding was that combination therapy significantly increased the risk for renal dysfunction compared with ramipril or telmisartan alone [8,9,10,11,12,13,14,15,16,17,18]. Most of the studies have used aliskiren as the study drug in patients with kidney diseases. Animal models show improved survival and decreased renal injury in animals receiving aliskiren. Human studies in patients with normal renal function show effective blood pressure control alone or in combination with different agents. In a randomized clinical trial, aliskiren decreased albuminuria when added to losartan in patients with early diabetic nephropathy. Ongoing studies are evaluating the role of renin inhibitors in the prevention of cardiovascular events and slowing of kidney disease progression in CKD. The results of AVOID suggest the renoprotective benefits of combination therapy extend to the direct renin inhibitors (DRI). In AVOID, combination therapy with aliskiren, a DRI, and losartan resulted in 20% greater protein excretion decrement than losartan monotherapy. Future trials should examine higher RAAS inhibitor doses, facilitate differentiation of renoprotective and antihypertensive effects of RAAS blockade, and use end-points that robustly demonstrate renoprotective effects [8,9,10,11,12,13,14,15,16,17,18,19,20,21,22]. In particular ALTITUDE will determine whether dual RAAS blockade with the direct renin inhibitor aliskiren in combination with an ACEi or ARB will reduce major morbidity and mortality in a broad range of high-risk patients with type 2 diabetes [23].

## Renin Inhibition: Should It Supplant ACE inhibitors and ARBS in High Risk Patients?

The potential advantages and disadvantages of aliskiren therapy *versus* existing RAAS antagonists in treating hypertension and target organ damage are under investigation. The antihypertensive efficacy of aliskiren monotherapy has been compared with that of other RAAS antagonists and combinations of aliskiren with these agents. Many studies have shown that aliskiren is equally effective as angiotensin receptor blockers and may be slightly more effective than angiotensin converting enzyme inhibitors in lowering blood pressure. In contrast to the other RAAS antagonists, aliskiren shuts down the entire downstream RAAS cascade. This results in greatly increased plasma renin concentration due to removal of angiotensin II-mediated feedback inhibition of renin release, which has raised concerns about whether direct renin inhibition adds anything to inhibition of downstream components of the RAAS cascade [24].

Comparative effects of aliskiren-based and ACE-based therapy on the renin system during long-term (6 months) treatment and withdrawal in patients with hypertension were compared in some study. Andersen *et al*., that compared DRI to ramipril 10 mg conclude that aliskiren-based therapy produced sustained blood pressure (BP) and PRA reductions over 26 weeks; ramipril-based therapy lowered BP and increased PRA. PRA reductions persisted four weeks after stopping aliskiren, suggesting an inhibitory effect beyond the elimination half-life of the drug. Palatini *et al*. reported that aliskiren 300 mg provided a sustained BP-lowering effect beyond the 24-h dosing interval, with a significantly smaller loss of BP-lowering effect in the 24-48 h period after dose than irbesartan 300 mg or ramipril 10 mg [10].

The effects of the direct renin inhibitor aliskiren were compared with losartan in patients with hypertension and left ventricular hypertrophy. In this report aliskiren was as effective as losartan in promoting LV mass regression. Reduction in LV mass with the combination of aliskiren plus losartan was not significantly different from that with losartan monotherapy, independent of blood pressure lowering. These findings suggest that aliskiren was as effective as an angiotensin receptor blocker in attenuating this measure of myocardial end-organ damage in hypertensive patients with LV hypertrophy. Finally DRI was compared with enalapril 20 mg. The effect is long-lasting and, at a dose of 160 mg, is equivalent to that of 20 mg enalapril, and the renin inhibitor aliskiren dose-dependently decreases Ang II levels in humans following oral administration [23,24,25,26].

## Combination Renin-Angiotensin System Blockade with Terenin Inhibitor Aliskiren in Hypertension

Combining an angiotensin-converting enzyme inhibitor and angiotensin II receptor blocker lowers blood pressure by 4/3 mmHg compared to either agent alone,although this additive effect may be abolished with maximal monotherapy dosing. The recent ONTARGET study showed no reduction in primary outcomes when an ACE-I-ARB combination was compared to an ACE-I alone, despite 2.4/1.4 mmHg lower BP in the former group. In proteinuric chronic kidney disease, an ACE-I-ARB combination reduces proteinuria and disease progression more than monotherapy, but the ONTARGET study showed an increase in renal endpoints in the combined group. Aliskiren offers a novel approach to renin-angiotensin system (RAS) inhibition. As monotherapy in hypertension, aliskiren is of similar efficacy to thiazides, calcium channel blockers and ARBs. In combination with other RAS inhibitors at maximal dosage aliskiren has a small synergistic effect on BP. Early data suggest a role for aliskiren in preventing end-organ damage but, considering the ONTARGET results with an ACE-I-ARB combination, outcome studies are needed before the use of aliskiren can be recommended in combination with other RAS inhibitors [5, 18,19,20,21,22,23,24,25,26,27,28,29,30]. 

Till now aliskiren was added to valsartan in stage 2 hypertension in a recent report. This combination therapy provided significantly greater BP reductions over aliskiren or valsartan monotherapy. Also aliskiren was combined with losartan, with similar results. Finally, Freiberger *et al*. tested the effect of a triple blockade of the renin-angiotensin-system in a patient affected by recurrent focal segmental glomerulosclerosis (FSGS) after kidney transplantation: they found a significant and sustained antiproteinuric effect under triple RAS blockade, and conclude that RAS blockade was generally well tolerated and can offer a new therapeutic approach in selected hypertensive patients with recurrent FSGS [31].

## Conclusions

Aliskiren has the potential to become the first orally active renin inhibitor that provides a true alternative to ACE-inhibitors and Ang II receptor antagonists in therapy for hypertension and other cardiovascular and renal diseases. As monotherapy, aliskiren now is reserved for use as an alternative to ACE-Is or ARBs, where these are ineffective or poorly tolerated. Till now a large, formal, and prospective randomized study with major endpoints with aliskiren has not been completed Without such data, especially when alternative therapeies have such data, it is difficult to make a firm recommendation. Aliskiren is well on its way but falls short of this criterion until the navigator study is complete. In our opinion in the future it is possible that aliskiren could supplant ACE inhibitors and ARBs, because it was demonstrated more effective than ACE and ARB to reduce BP and to reduce PRA. Combination therapy with the direct renin inhibitor, aliskiren, and the angiotensin receptor blocker or ACE inhibitor has been shown to produce greater BP reductions than either agent alone in some double-blind study and it will represent the next future.
