# Novo, S. *et al.* Aliskiren: Just a New Drug for Few Selected Patients or an Innovative Molecule Predestinated to Replace Arbs and Ace-Inhibitors? *Pharmaceuticals* 2009, *2*, 118-124

**DOI:** 10.3390/ph4101293

**Published:** 2011-09-30

**Authors:** Salvatore Novo, Giovanni Fazio, Elena Raccuglia, Antonino Mignano, Giuseppina Novo

**Affiliations:** Department of Internal Medicine and Cardiovascular Diseases, University Hospital “Paolo Giaccone”, University of Palermo, Italy

We found following mistakes in our paper published in Pharmaceuticals [1]:

In the published version “Paladini *et al*. reported that aliskiren 300 mg provided a sustained BP-lowering effect beyond the 24-h dosing interval, with a significantly smaller loss of BP-lowering effect in the 24-48 h period after dose than irbesartan 300 mg or ramipril 10 mg [25]”. Paladini *et al*. should be Palatini *et al*., and the cited reference number should be [10], not [25]..

In the sentence, “Early data suggest a role for aliskiren in preventing end-organ damage but, considering the ONTARGET results with an ACE-I-ARB combination, outcome studies are needed before the use of aliskiren can be recommended in combination with other RAS inhibitors [18–30]” one more citation number was added [5], so the revised sentence is “Early data suggest a role for aliskiren in preventing end-organ damage but, considering the ONTARGET results with an ACE-I-ARB combination, outcome studies are needed before the use of aliskiren can be recommended in combination with other RAS inhibitors [5,18–30]”.

The references [24] to [31] are missing from the published version. They are listed below:

24. McMurray, J.J.; Pitt, B.; Latini, R.; Maggioni, A.P.; Solomon, S.D.; Keefe, D.L.; Ford, J.; Verma, A.; Lewsey, J. Effects of the oral direct renin inhibitor aliskiren in patients with symptomatic heart failure.*Circ. Heart Fail.* **2008**, *1*, 17-21.

25. Solomon, S.D.; Appelbaum, E.; Manning, W.J.; Verma, A.; Berglund, T.; Lukashevich, V.; Cherif-Papst, C.; Smith, B.A.; Dahlöf, B. Effect of the direct Renin inhibitor aliskiren, the Angiotensin receptor blocker losartan, or both on left ventricular mass in patients with hypertension and left ventricular hypertrophy. *Circulation* **2009**, *119*, 530-537.

26. Doulton, T.W.; MacGregor, G.A. Combination renin-angiotensin system blockade with the renin inhibitor aliskiren in hypertension. *J. Renin. Angiotensin. Aldosterone Syst.* **2009**, *10*, 185-189.

27. Baldwin, C.M.; Plosker, G.L. Aliskiren/hydrochlorothiazide combination: in mild to moderate hypertension. *Drugs* **2009**, *69*, 833-841.

28. Pimenta, E.; Oparil, S. Role of aliskiren in cardio-renal protection and use in hypertensives with multiple risk factors. *Vasc. Health Risk Manag.* **2009**, *5*, 453-463.

29. Andersen, K. Renin-angiotensin-aldosterone system in the elderly: rational use of aliskiren in managing hypertension. *Clin. Interv. Aging* **2009**, *4*, 137-151.

30. Mann, J.F.; Schmieder, R.E.; McQueen, M.; Dyal, L.; Schumacher, H.; Pogue, J.; Wang, X.; Maggioni, A.; Budaj, A.; Chaithiraphan, S.; Dickstein, K.; Keltai, M.; Metsärinne, K.; Oto, A.; Parkhomenko, A.; Piegas, L.S.; Svendsen, T.L.; Teo, K.K.; Yusuf, S. Renal outcomes with telmisartan, ramipril, or both, in people at high vascular risk (the ONTARGET study): a multicentre, randomised, double-blind, controlled trial. *Lancet* **2008**, *372*, 547-553.

31. Freiberger, V.; Amann, K.; Heemann, U.; Frank, H Effect of a triple blockade of the renin-angiotensin-system in recurrent focal segmental glomerulosclerosis after kidney transplantation. *Transpl. Int.* **2009**, *22*, 1110-1113.
